# SEQdata-BEACON: a comprehensive database of sequencing performance and statistical tools for performance evaluation and yield simulation in BGISEQ-500

**DOI:** 10.1186/s13040-019-0209-9

**Published:** 2019-11-15

**Authors:** Yanqiu Zhou, Chen Liu, Rongfang Zhou, Anzhi Lu, Biao Huang, Liling Liu, Ling Chen, Bei Luo, Jin Huang, Zhijian Tian

**Affiliations:** BGI-Wuhan Clinical Laboratories, Building B2, No.666 Gaoxin Road, Wuhan East lake Hi-tech Development zone, Wuhan, 430074 China

**Keywords:** BGISEQ-500, Sequencing run performance, Prediction tools, Data analysis, Database

## Abstract

**Background:**

The sequencing platform BGISEQ-500 is based on DNBSEQ technology and provides high throughput with low costs. This sequencer has been widely used in various areas of scientific and clinical research. A better understanding of the sequencing process and performance of this system is essential for stabilizing the sequencing process, accurately interpreting sequencing results and efficiently solving sequencing problems. To address these concerns, a comprehensive database, SEQdata-BEACON, was constructed to accumulate the run performance data in BGISEQ-500.

**Results:**

A total of 60 BGISEQ-500 instruments in the BGI-Wuhan lab were used to collect sequencing performance data. Lanes in paired-end 100 (PE100) sequencing using 10 bp barcode were chosen, and each lane was assigned a unique entry number as its identification number (ID). From November 2018 to April 2019, 2236 entries were recorded in the database containing 65 metrics about sample, yield, quality, machine state and supplies information. Using a correlation matrix, 52 numerical metrics were clustered into three groups signifying yield-quality, machine state and sequencing calibration. The distributions of the metrics also delivered information about patterns and rendered clues for further explanation or analysis of the sequencing process. Using the data of a total of 200 cycles, a linear regression model well simulated the final outputs. Moreover, the predicted final yield could be provided in the 15th cycle of the early stage of sequencing, and the corresponding R^2^ of the 200th and 15th cycle models were 0.97 and 0.81, respectively. The model was run with the test sets obtained from May 2019 to predict the yield, which resulted in an R^2^ of 0.96. These results indicate that our simulation model was reliable and effective.

**Conclusions:**

Data sources, statistical findings and application tools provide a constantly updated reference for BGISEQ-500 users to comprehensively understand DNBSEQ technology, solve sequencing problems and optimize run performance. These resources are available on our website http://seqBEACON.genomics.cn:443/home.html.

## Background

Next generation sequencing (NGS, also known as high-throughput sequencing) has led us into the genomic era. In the past 15 years, the development of sequencing technology has been mainly committed to a reduction in cost and improvements in throughput, accuracy and read lengths. Currently, the sequencer manufacturers Illumina and Beijing Genomics Institute (BGI) provide high throughput and accuracy, while Pacific Bioscience and Oxford Nanopore offer long read lengths [1, 2]. The first BGI sequencer BGISEQ-500 was launched in 2015 (http://en.mgitech.cn/), which was based on two key technologies: DNA nanoball (DNB) and Combinatorial Probe-Anchor Synthesis (cPAS). In library preparation, DNA is fragmented, end-repaired and ligated with adapters. The ligation product is amplified by PCR for several cycles to become DNA libraries. These libraries are then circularized by DNA ligase with a splint oligo that was reverse complemented to one strand. Next, the DNA library circles are replicated with polymerase *phi29* to form a single-stranded DNA molecule called a DNB by rolling circle amplification (RCA). The DNBs are dispersed and immobilized on a photolithographically etched, patterned flow cell by a loader machine. When sequencing, a probe is first annealed to a DNA molecular anchor on the DNB. In each cycle, DNA polymerase incorporates one base labeled with a fluorescence group, and the four colored light signals emitted by the bases are collected via a high-resolution imaging system and converted into bases after basecalling [3].

Recently, BGISEQ-500 platform has been widely used in a variety of sequencing applications, such as whole genome sequencing (WGS) [4], whole exome sequencing (WES) [5], RNA-seq [6], small RNA and metagenomics [7, 8]. BGISEQ-500 sequencing platform has not only participated in the transcriptome analysis of plant nitrogen metabolism [9] but also in human clinical applications for cancer genome sequencing and TP53 mutation detection in high-grade serous ovarian cancer [10, 11]. In addition, it has been reported that BGISEQ-500 has good performance at the single-cell resolution, such as in scRNA-seq and scCAT-seq [12–14]. Compared with the Illumina platforms, the BGISEQ-500 is cost-effective and has low error and duplication rates [1, 13]. The throughput per lane of BGISEQ-500 is approximately twice as HiSeq4000 in the PE100 sequencing type. The cost per gigabase (Gb) is 40–60% of that of the Illumina HiSeq4000 platform. The generation of DNBs is based on rolling circle amplification, which effectively prevents errors from PCR amplification. The sequencing data derived by BGISEQ-500 are compatible with widely used bioinformatics tools and pipelines such as GATK, bwa, HISAT, DEseq2 and SnpEff [15]. The only difference is the setting of some software parameters. To date, the results obtained from data generated from Illumina and BGISEQ-500 platforms have been comparable. For example, BGISEQ-500 demonstrates comparable SNP detection accuracy in WGS [10], similar consistency in variation detection in WES [5], and high concordance in transcriptome and metagenomic studies [16, 17]. Therefore, DNBSEQ technology provides a new choice for resolving issues in scientific research and agriculture, environment and clinical applications.

The performance of sequencers is very important for the throughput, quality and reliability of data generated in each run. Among the sequencing performance metrics in BGISEQ-500, yield (e.g., Reads, the number of DNBs recognized by the Basecall software) and quality (e.g., Q30, the percentage of bases with an error rate below 0.001) are of the highest concern. Other metrics regarding chemical reaction and instrument state are also recorded in the sequencing summary. However, we still lack a profound understanding of these metrics, especially the connections between them. As one of the world’s largest sequencing service providers, BGI performs thousands of sequencing runs each year. Accompanied with enormous amounts of nucleotide data, massive sequencing performance data are highly valuable for illustrating this complicated process and for troubleshooting. Unfortunately, this type of datasets or databases are presently rare, and a comprehensive database is required to integrate the abundant run performance data.

In this study, we designed a database, SEQdata-BEACON, to comprehensively collect sequencing performance data from BGISEQ-500, including sample, yield, quality, machine state and supplies. We calculated Pearson’s correlation coefficients for 52 numerical metrics for hierarchical clustering and analyzed their distribution patterns. We also used linear regression to establish yield simulation models to investigate the connections among yield correlated metrics and attempted to predict the final yield at the early stage of sequencing. All the data and statistical analysis results are available on our open access website. These resources can be used as an updating reference dataset for BGISEQ-500 users in different enterprises or schools to gain a deeper understanding of DNBSEQ technology.

## Methods

### Data collection and database construction

DNA libraries were loaded onto a patterned array. After successive chemical reactions, signal acquisition and basecalling, the sequencers normally generate a series of folders and files in each cycle or at the end of the sequencing process. In BGISEQ-500, more than 10 files recorded the run configuration data of the entire sequencing process, such as InputInfo_*.txt, RunInfo.txt, summaryReport.html, fovReport.QC.txt, BarcodeStat.txt and fq.fqStat.txt. We chose 60 BGISEQ-500 sequencers in the BGI-Wuhan lab and collected all available files generated following chemical reactions and basecalling. The criteria for selecting metrics from these files are as follows: 1) metrics of great concern and closely related to the sequencing process [18]; 2) metrics covering information on the sequencing type, the optical path state of the machine etc.; 3) metrics related to traceable information for troubleshooting. Based on these, we used flow cell identifier (FC) as an index to extract 64 metrics from data resources to create a database ‘SEQdata-BEACON’. Each entry was assigned a unique identification number (ID). We accumulated lanes in paired-end 100 (PE100) sequencing since it is the major sequencing type in BGISEQ-500. No detailed sample information was entered into the database in order to protect the privacy of customers. The database was constructed on a MySQL server (version 8.0).

### Web visual interface and statistical analysis

A user-friendly interface for SEQdata-BEACON was built on Apache (version 2.4.33 win64 VC15 server). The Google Chrome web browser (version 68.0.3440.106) is suggested to access the website. Statistical analysis and figures in this study were generated based on data obtained from our database with R software (version 3.5.0 x 64) with the ability to install additional packages as needed.

### Yield simulation model

We used yield-related independent variables to construct the yield simulation model. First, considering the sequencing principle, DNB was the definitive source for yield. The number of successfully fixed DNBs on the patterned array determined the ability to produce reads [3, 18]. TotalEsr (Total Effective Spot Rate) multiplied by Dnbnumber excluded those spots without DNBs or with dark DNBs that showed no light signal and represented the maximum signal that could be successfully collected. Second, metrics that changed with the sequencing process were also included, such as BIC (Basecall information content), accGRR (Accumulated Good Reads Rate), SNR (Signal to Noise Ratio), FIT (indicates the distribution of differences between signal and noise for each base), Intensity (Light intensity of each base (ATCG) for each cycle), Runon and Lag (the percentage of read strands being out of phase with the current cycle). These metrics reflect the loss in final yield resulting from chemical reactions, light intensity, camera acquisition and basecalling in the sequencing process. The loss in yield is primarily composed of filtered reads. Third, the values of these metrics were collected from our database as the datasets. The correlations of each metric with yield were studied, and the ones that were linearly correlated with yield were chosen (Additional file 1: Figure S1). We used Tukey’s boxplot to pinpoint the possible outliers, which were defined as observations that fell below Q_1_–1.5 IQR (interquartile range) or above Q_3_ + 1.5 IQR and excluded from the model construction. Finally, we constructed a linear regression (LR) model with TotalEsr*Dnbnumber, BIC, accGRR, SNR and FIT as input variables and yield (Reads) as the output variable. The LR model formula was as follows (Eq. (1)):

1 $$ Y={\beta}_0+{\beta}_1 TD+{\beta}_2B+{\beta}_3G+{\beta}_4S+{\beta}_5F+\varepsilon, \upvarepsilon \sim \mathrm{Normal}\left(0,\upsigma \right) $$

*Y* is the value of the dependent variable yield. *T* is the value corresponding to the current cycle in TotalEsr. *D* is the value of Dnbnumber at the beginning of the sequencing. *B*, *G*, *S* and *F* are the average values of BIC, accGRR, SNR and FIT, respectively, for the first 200 cycles. ε is the observed error and obeys a normal distribution. The model parameters *β*_*n*_ are the coefficient values estimated using a regression model.

Furthermore, we simulated the final yield based on the metric values obtained every 5 cycles. The formula was as follows (Eq. (2)):

2 $$ {Y}_i={\beta}_{0,i}+{\beta}_{1,i}{T}_i{D}_i+{\beta}_{2,i}{B}_i+{\beta}_{3,i}{G}_i+{\beta}_{4,i}{S}_i+{\beta}_{5,i}{F}_i+{\varepsilon}_i,{\varepsilon}_i\sim \mathrm{Normal}\left(0,\upsigma \right) $$

*Y*_*i*_ is the value of the dependent variable yield. *T*_*i*_ is the value corresponding to the current cycle i in TotalEsr. *D*_*i*_ is the value of Dnbnumber at the beginning of the sequencing. *B*_*i*_, *G*_*i*_, *S*_*i*_ and *F*_*i*_ are the average values of BIC, accGRR, SNR and FIT, respectively, for the first i cycles. *ε*_*i*_ is the observed error in the i cycle and obeys a normal distribution. The model parameters *β*_*n*, *i*_ are the coefficient values estimated using a regression model. The coefficient of determination (R^2^), which ranges from 0 to 1, was used to measure the accuracy of our model; a value closer to 1 means better performance of the model.

Evaluation of the linear regression model was performed with test sets to test the reliability of the prediction model. The test sets were used in the model formula to obtain prediction results. An R^2^ for the test sets was used to evaluate the constructed model. Both linear regression and the backward elimination method of stepwise regression and evaluation were conducted in R.

## Results

### Data collection and database construction

**Fig. 1:**
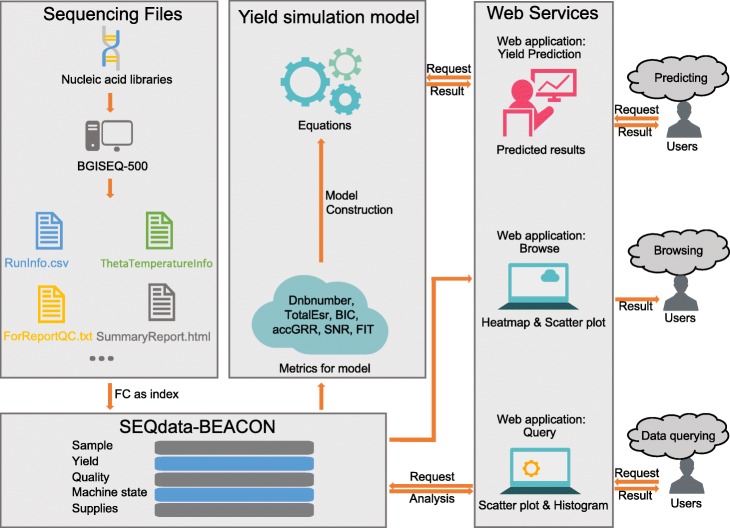
Schematic architecture of SEQdata-BEACON. The architecture of the database and applications contains four parts. “Sequencing Files” shows the files generated from BGISEQ-500 sequencers. “SEQdata-BEACON” shows the information of metrics. “Yield simulation model” shows the special function of our database to predict the final yield from input metrics. “Web services” shows three applications in our website to provide interactive functions for users--Predicting, Browsing and Data querying

The DNA sequencing process generated a series of files used to record sequencing performance information. In this study, we extracted 65 metrics to construct a database ‘SEQdata-BEACON’ to store the massive data source. The architecture of the database and applications is shown in Fig. 1. From November 2018 to April 2019, ‘SEQdata-BEACON’ accumulated a total of 2236 entries in PE100 sequencing using a 10 bp barcode from 60 sequencers in the BGI-Wuhan lab. The database primarily collected information on sample, yield, quality, machine status and supplies. Sequencing type and other sample information were stored as Sample. Yield and quality are the gold standards of the reliability of our sequencing data. Regular quality control metrics such as Reads, Bases—related to yield and Q30—related to quality were stored. Machine performance reflects the stable environment of the sequencing process, so machine states such as Signal, Intensity and Theta were stored in the database. Supplies provides traceable information to solve problems in troubleshooting, so sequencing reagents and sequencing time were also stored in the database. Furthermore, we explored our database to describe the statistical results of metric features and to construct a yield simulation model based on yield-related metrics.

### Web visual interface

**Fig. 2:**
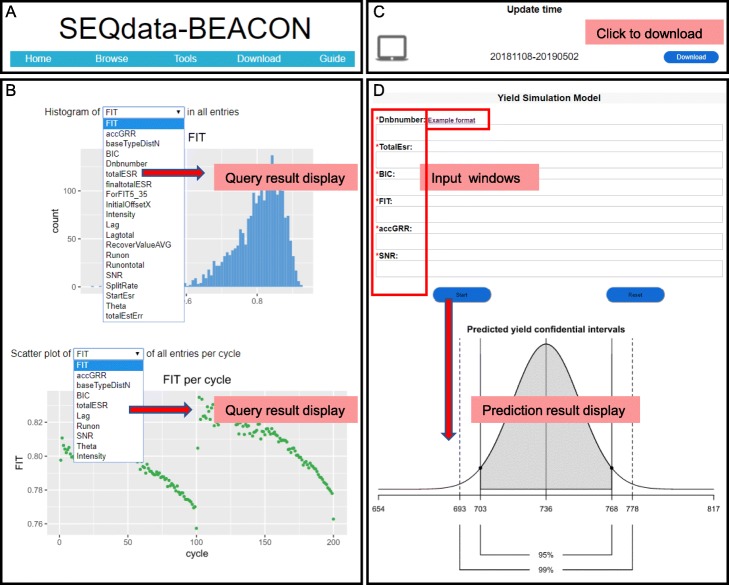
Web visual interface of SEQdata-BEACON. The screen shots show views of functional modules. **a** The navigator bar on the “Home” page. **b** The drop-down menu and distribution charts of metrics are shown on the “Browse” page. **c** The download sources are listed on our website’s “Download” page. **d** The input windows and the results of the yield simulation model are shown on the “Tools” page. Please note that not all fields are shown

To provide open access to our data, we designed a comprehensive website ‘SEQdata-BEACON’ with Home, Browse, Tools, Download and Guide pages to display the database and data-mining applications (Fig. 2a). The ‘Home’ page gives users an introduction to our website and a schematic architecture of our database. The ‘Browse’ page allows users to look through the numerical metric features, including a heatmap of Pearson’s correlation coefficients and the metric distributions. For example, the distribution of FIT and its changes per cycle are both illustrated in charts for observing the distribution patterns and fluctuations. Users can choose the name of their metric of interest in the drop-down menu, and the corresponding distribution chart will be shown at the bottom of the webpage (Fig. 2b; see the section ‘Statistical findings: metric features’ for details). The ‘Download’ page allows users to obtain the data in EXCEL format according to our update time; all the data and analysis results will be updated every 2 months (Fig. 2c). The ‘Tools’ page allows users to test our simulation model. Users can enter specific metric values on our website in the example format and click ‘Start’, then the expected yield confidential intervals will be shown (Fig. 2d; see the section ‘Statistical findings: yield simulation model’ for details). The ‘Guide’ page supplies a guideline for regular operation.

### Statistical findings: metric features

**Fig. 3:**
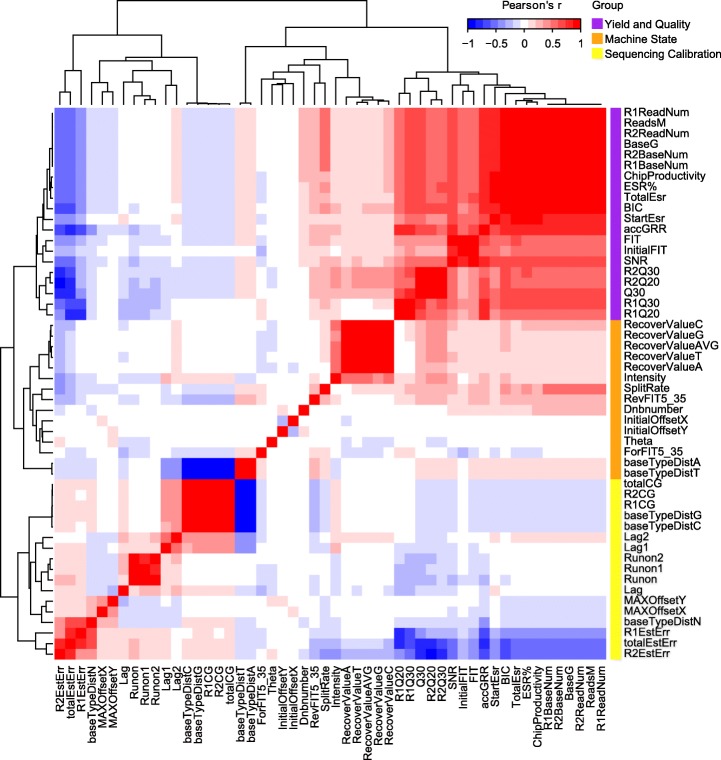
Numerical metrics correlation. Hierarchical clustering of the Pearson’s correlation matrix between 52 metrics. The three branches Yield and Quality, Machine State, and Sequencing Calibration are marked with purple, orange and yellow, respectively, on the right side of the Y-axis in the figure. Red blocks in the heatmap indicate positive relationship, blue blocks indicate negative relationship, and white blocks indicate no relationship

Based on the data from November 2018 to April 2019, Pearson’s correlation coefficients of the numerical metrics were calculated, and the resulting 52*52 correlation matrix is shown as a heatmap (Fig. 3). The metrics were mainly clustered into three groups with 20, 15 and 17 metrics; the first group could be further divided into two branches that represent yield and quality. The other two groups represent machine state (e.g., Theta indicates the angle between the moving direction of the array stage and the track line on the array) and sequencing calibration (e.g., Lag, Runon). Red blocks indicate positive relationship and blue blocks indicate negative relationship. Yield was positively correlated with quality, and the sequencing calibration was negatively correlated with both. It can also be seen that five metrics with a Pearson correlation of 1 with Reads were redundant; these were deleted from further analysis. In addition, totalCG% (the percent of CG based in sequencing lane) is a conceptual summary of R1CG% and R2CG%, and only one metric was reserved according to analytical needs.

**Fig. 4:**
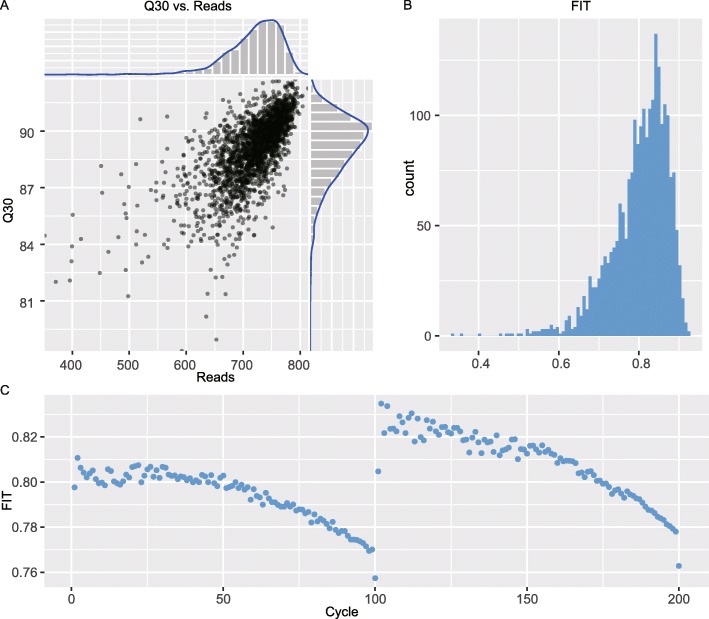
Distribution of Metrics in SEQdata-BEACON. **a** Q30 versus Reads, histogram of Q30 and Reads shown in gray, density profiles shown in blue. **b** Histogram of FIT in all entries. **c** Scatterplot of FIT through 200 cycles

Next, we investigated the distribution patterns of the above metrics. It can be seen in the scatterplot of Q30 versus Reads that yield ranged from 350 to 850 M, and Q30 was above 70%. The histograms above and to the right of the chart also show the distributions of Reads and Q30, respectively (Fig. 4a). During normal performance of BGISEQ-500, Reads is greater than 650 M and Q30 is over 85% in each lane (http://en.mgitech.cn/). In the scatter plot, a total of 2026 lanes were located in the normal range, and the proportion of outliers was less than 10%, which suggests that the instrument performance was relatively stable. The distribution pattern of FIT was also plotted in a histogram, and it was mostly around 0.80 (Fig. 4b). The FIT value was calculated cycle by cycle, and it was found to slowly decrease from 0.811 to 0.757 in read1 and from 0.835 to 0.763 in read2, with more deviation at the beginning of both reads and less deviation toward the end (Fig. 4c). The metric FIT suggested the distribution of differences between signal and noise for each base and the performance of the optical path during the sequencing process [18]. The deviation in each cycle may reflect the changing pattern of FIT in the sequencing process and indirectly hint at the status of the signal or optical path.

### Statistical findings: yield simulation model

**Fig. 5:**
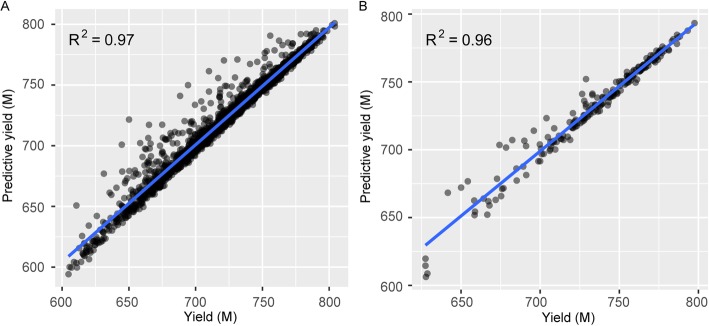
A comparison of predicted vs. actual yield in training sets and test sets. **a** Training sets. **b** Test sets. The linear regression line is shown in blue

In this study, considering the BGISEQ-500 sequencing principle, the meanings of the metrics and the linear correlations, the linear regression model included the variables TotalEsr, Dnbnumber, BIC, accGRR, SNR and FIT as predictors. Statistical program R was applied to construct the model using training sets obtained from November 2018 to April 2019 and to test the model with test sets obtained from May 2019. The LR model is shown in Eq. (3) and Table 1 displays the standard error, *t*-value and *p*-value of this function in the regression results. The results indicate that the contribution of accGRR was not significant. Subsequently, the backward elimination method of stepwise regression was used to build the model to find an optimum solution, and the final LR model is shown in Eq. (4). The regression results of this function indicate that the contributions of TotalEsr*Dnbnumber, BIC, SNR, FIT were significant at the 1% probability level (Table 1). Here, we compared the yield with the predicted results in our model, obtaining an R^2^ of 0.97. Then, the model was run with the test sets to predict the yield, which resulted in an R^2^ of 0.96. The performance of the real test meets the expectations of constructed model. These results indicate that our simulation model was reliable and effective (Fig. 5). It also suggests that five metrics, TotalEsr*Dnbnumber, BIC, SNR, FIT, successfully simulated yield in the LR model. In addition, we expected that this simulation model could be used to predict the final yield at the early stage of sequencing. During the sequencing process, the yield was assumed to be a function of the values of TotalEsr, Dnbnumber, BIC, accGRR, SNR and FIT at each running cycle. The model was run every 5 cycles using backward elimination regression with R, and a total of 40 model runs were performed from cycle 1 to 200. The residuals of the prediction models fluctuated at the beginning of read1 and read2, which was mainly due to the establishment of the algorithm matrix by the sequencer (Additional file 2: Figure S2). However, the R^2^ of the model in the 15th cycle was 0.81, indicating that the final yield could be effectively simulated at this stage. The corresponding LR model is shown in Eq. (5).

3 $$ Y=-129.692+0.983 TD+1.400B+0.143G-3.334S+26.997F $$

4 $$ Y=-129.787+0.983 TD+1.402B-3.334S+27.027F $$

5 $$ {Y}_{15}=-141.021+1.270 TD+4.743B-604.450G-3.465S+28.882F $$

**Table 1:** Model summaries of linear regressions for predicting yield outputs

Independent variables	Analytical results for Eq. (3)			Analytical results for Eq. (4)			Analytical results for Eq. (5)		
	Std. error	*t*-Value	*P*-value	Std. error	*t*-Value	*P*-value	Std. error	*t*-Value	*P*-value
Constants	11.424	−11.353	< 2e-16**	10.662	−12.173	< 2e-16**	39.411	−3.578	0.00035**
TotalEsr*Dnbnumber	0.010	95.192	< 2e-16**	0.010	96.130	< 2e-16**	0.033	38.837	< 2e-16**
BIC	0.192	7.278	4.73e-13**	0.178	7.873	5.46e-15**	0.724	6.556	6.91e-11**
accGRR	6.175	0.023	0.981	–	–	–	59.009	− 10.243	< 2e-16**
SNR	0.388	−8.586	< 2e-16**	0.388	−8.596	< 2e-16**	0.653	−5.307	1.23e-07**
FIT	4.757	5.675	1.57e-08**	4.577	5.905	4.08e-09**	11.249	2.567	0.010*

TotalEsr: ESR (Effective Spot Rate), the percentage of filtered Reads among the DNBs recognized by Basecalling. ESR = Total Reads/theoretical maximum reads number of one sequencing lane. TotalEsr calculated ESR value in the first 15 cycles in read1 and read2, and kept constant in the rest of each read

Dnbnumber: The theoretical maximum number of DNBs on the patterned array

BIC: Basecall information content, the percentage of DNBs that can be used for Basecalling among the DNBs recognized by the optical system. BIC = (numbers of DNB that can be used for Basecalling/numbers of DNB that can be recognized by the optical systems) × 100%

accGRR: Accumulated Good Reads Rate, taking chastity greater than 0.6 as the filtering criteria, the percentage of filtered Reads among the DNBs recognized by Basecalling. accGRR = Total Reads/theoretical maximum reads number of one sequencing lane. This value is only a statistical indicator which reflects the overall quality of the read (multi-cycle state)

SNR: Signal to Noise Ratio, taking the SNR calculation of a single DNB as an example, base A (maximum light intensity) is used as the signal, the CGT is the background, and the variance of the CGT light intensity is noise. A_SNR = A_mean/CGT_dev

FIT: FIT value represents the distribution of differences between signal and noise for each base. The FIT value is higher when the distribution of differences between signal to noise for each channel/color are more concentrated

**Significant at the 1% probability level

* Significant at the 5% probability level

## Discussion

Continuous improvement in DNBSEQ technology has introduced more efficient sequencing platforms, such as MGISEQ-2000 and DNBSEQ-T7. Compared to the Illumina platform, BGISEQ-500 is cheap and has a low sequencing error rate. Sequencing costs are often affected by geographic, institutional, personnel, and reagent costs, and continue to decline as technology updates. According to recent research statistics [13], taking the PE100 sequencing type as an example and not concerning the physical loss of the sequencer, the cost per Gb for the BGISEQ-500 is half that of the HiSeq4000 platform. And BGISEQ-500 sequencing data showed a lower error rate than Illumina (< 0.1 and 0.1%, respectively) [1]. Moreover, the sequencing data produced from BGISEQ-500 are compatible with widely used bioinformatics tools and pipelines. The data analysis results showed comparable accuracy and reproducibility. Recent investigations have reported that MGISEQ-2000 has comparable single nucleotide polymorphism (SNP) detection accuracy in WGS and high gene detection in scRNA-seq to Illumina platforms [19, 20].

In fact, the stable performance of sequencers in massively parallel sequencing guarantees the utility of data and the cost efficiency of each run. Some tools have been reported to evaluate sequencing run performance by analyzing sequencing data quality. For example, FASTQC is a commonly used tool for quality control of sequencing data and the generation of a comprehensive QC report [21]. It can also be incorporated into an analysis pipeline to represent the quality of raw data in easy-to-browse HTML reports [22]. The whole NGS workflow included library and template preparation, enrichment, sequencing and data analysis, but quality control (QC) checkpoints for sequencing performance were often performed in the data quality check rather than in the sequencing process [23]. Different from data quality evaluation, the run performance metrics brought us a wealth of information that could be used to effectively assess the sequencing process and its results. To gain insight into the sequencing performance in BGISEQ-500, we established the first-reported BGISEQ-500 sequencing performance database and website to comprehensively collect performance data.

There were 2236 entries with 65 metrics containing information on sample, yield, quality, machine state and supplies in ‘SEQdata-BEACON’. The method of automatically collecting metric values from sequencing configuration files could effectively lighten human labor, shorten time costs and improve data accuracy. The run data we collected covered libraries from most species and major types of sequencing applications. At present, in our 60 BGISEQ-500 sequencers in the BGI-Wuhan lab, PE100 sequencing using a 10 bp barcode is suitable for WGS, WES and RNA-seq, and the libraries are derived from DNA or RNA samples of plants, animals, microbes and humans. In the Q30 versus Reads scatterplot, 90.6% of the lanes had reads greater than 650 M and values of Q30 above 85%, which shows that BGISEQ-500 was stable and reliable in massively parallel sequencing. Therefore, without the risk of index hopping, DNBSEQ can generate excellent sequencing data with fewer duplications and errors [24] and has extensive application in population-scale sequencing projects, such as the 10KP (10,000 Plants) Genome Sequencing Project [25]. To study the correlation of yield-associated metrics, we used the backward elimination method of stepwise regression and established a yield simulation model with an R^2^ of 0.97. The model produced a good simulation, which suggests that TotalEsr, Dnbnumber, BIC, SNR and FIT contributed to the yield. While predicting the final production, we used all six parameters to construct 40 prediction models using stepwise regression. From the residual deviation of the models, it was shown that the final yield could be predicted in the 15th cycle at the early stage of sequencing, and the small changes in the residuals within read1 and read2 implied little fluctuation of the metrics during the sequencing process. The linear regression model is a common statistical technique for simulating the associations between variables, but whether other methods may produce better simulation results cannot be ruled out. Furthermore, we wanted to investigate quality-associated metrics and establish a quality simulation model. Combined with the yield simulation model, these two models may effectively simulate the sequencing results and bring us more ideas for increasing the sequencing performance.

Recently, the sequencer manufacturer Illumina revealed a new service named “Proactive Instrument Monitoring”, which is a proactive support service that involves remote instrument monitoring in real time [26]. By sending instrument performance data to Illumina, the support team can monitor the instrument and resolve issues more quickly. Apart from monitoring the instrument performance, our study paid more attention to data accumulation and was expected to explore data patterns by statistical analysis and interpret sequencing results. In the future, we plan to gather more sequencing platforms built on DNBSEQ technology, which will provide an integrated performance reference for BGI sequencers and will be beneficial to fully understand this series of instruments. Moreover, we also want to add the PacBio Sequel II and Oxford Nanopore PromethION sequencers to obtain a deeper understanding of single-molecule sequencing technology. We expect SEQdata-BEACON to be a comprehensive platform: with data accumulation, it can demonstrate the actual performance of the sequencing platforms; by developing more data-mining applications, it can enrich functional tools such as QC metrics models and metrics standards; by presenting data and statistical results on the website, it can also give users useful optimization and troubleshooting suggestions to solve their problems.

## Conclusions

Widespread application of NGS has resulted in a large amount of data, including nucleotide sequences and sequencing process performance. We designed a database, SEQdata-BEACON, to accumulate run performance data from BGISEQ-500 containing 65 metrics with information on sample, yield, quality, machine state and supplies. A correlation matrix of 52 numerical metrics was clustered into three groups: yield-quality, machine state and sequencing calibration. The distribution of numerical metrics presented some features and provided clues for further interpreting the meanings of these metrics and their analysis. We also constructed linear regression models to accurately simulate the final yield using metric values in the 200th and 15th cycles of the runs. The data sources, statistical findings and application tools are all available on our website (http://seqBEACON.genomics.cn:443/home.html), which can facilitate BGISEQ-500 users from enterprises or schools to understand DNBSEQ technology and interpret their sequencing results.

## Supplementary information

**Additional file 1:** **Figure S1.** The correlation of each metric with yield (ReadsM). The linear regression line is shown in blue.

**Additional file 2:** **Figure S2.** Residual deviation of the LR model every 5 cycles. The box plot displays the residuals of all 40 LR models; each box shows the median and first and third quartiles, and a star indicates the standard deviation.

## Data Availability

The datasets generated and/or analysed during the current study are available in the SEQdata-BEACON site, [http://seqBEACON.genomics.cn:443/home.html].

## Abbreviations

accGRR: Accumulated Good Reads Rate
BGI: Beijing Genomics Institute
BIC: Basecall information content
cPAS: Combinatorial Probe-Anchor Synthesis
DNB: DNA nanoball
Eq.: Equation
FC: Flow cell identifier
Gb: Gigabase
ID: Identification number
IQR: Interquartile range
LR: Linear regression
NGS: Next generation sequencing
PE100: Paired-end 100
QC: Quality control
RCA: Rolling circle amplification
scCAT-seq: Single-cell chromatin accessibility and transcriptome sequencing
scRNA-seq: Single cell RNA sequencing
SNP: Single nucleotide polymorphism
SNR: Signal to Noise Ratio
TotalEsr: Total Effective Spot Rate
WES: Whole exome sequencing
WGS: Whole genome sequencing
