# Supplementary figures and images for: SEQdata-BEACON: a comprehensive database of sequencing performance and statistical tools for performance evaluation and yield simulation in BGISEQ-500
Source: BioData Min. 2019 Nov 15;12:21. doi: 10.1186/s13040-019-0209-9 (PMC6857306; doi:10.1186/s13040-019-0209-9)

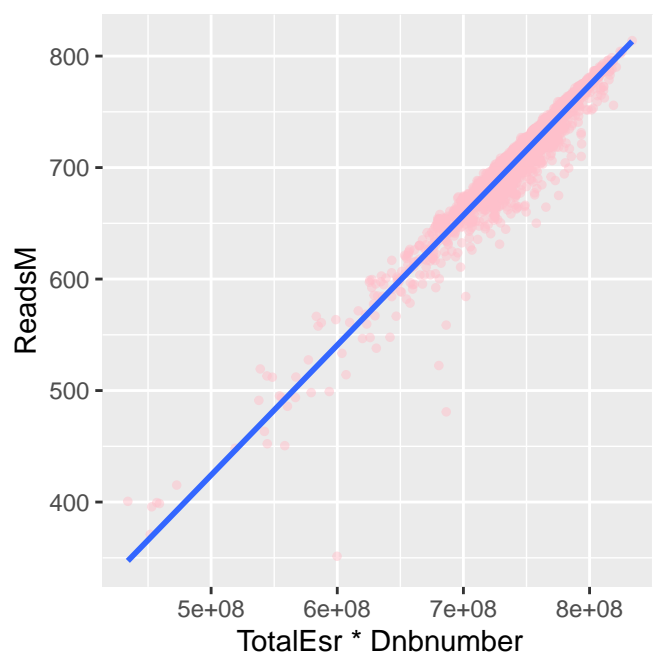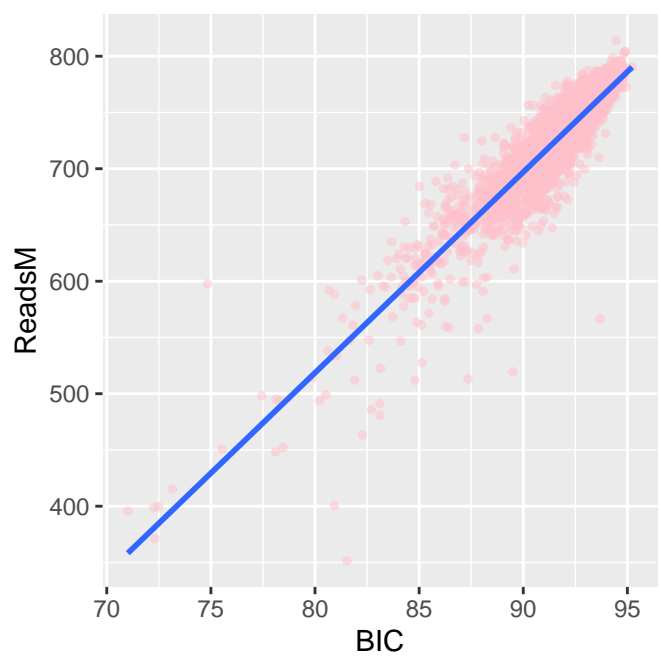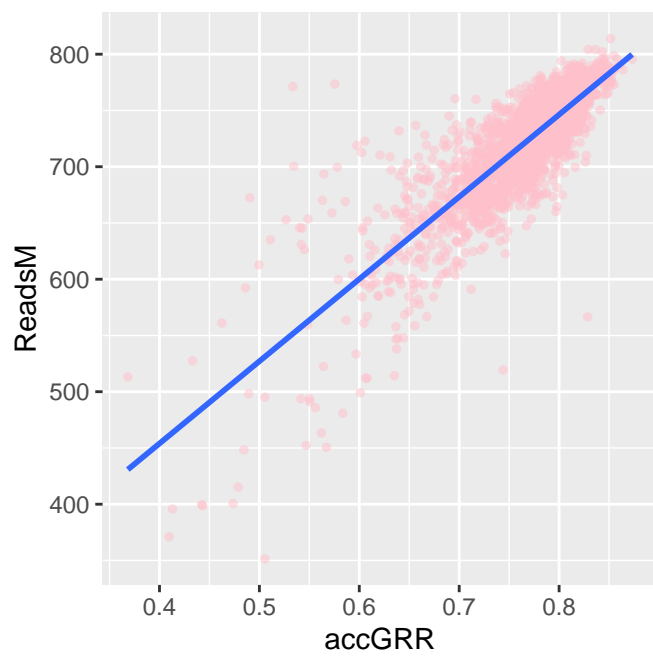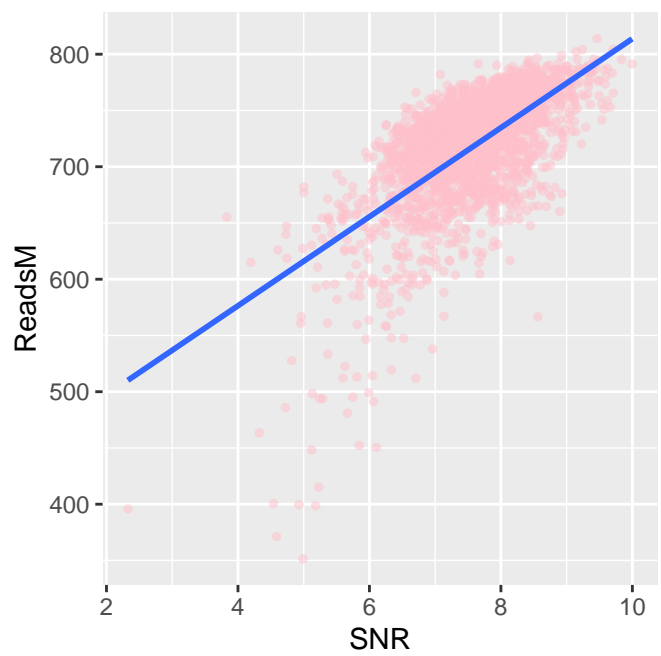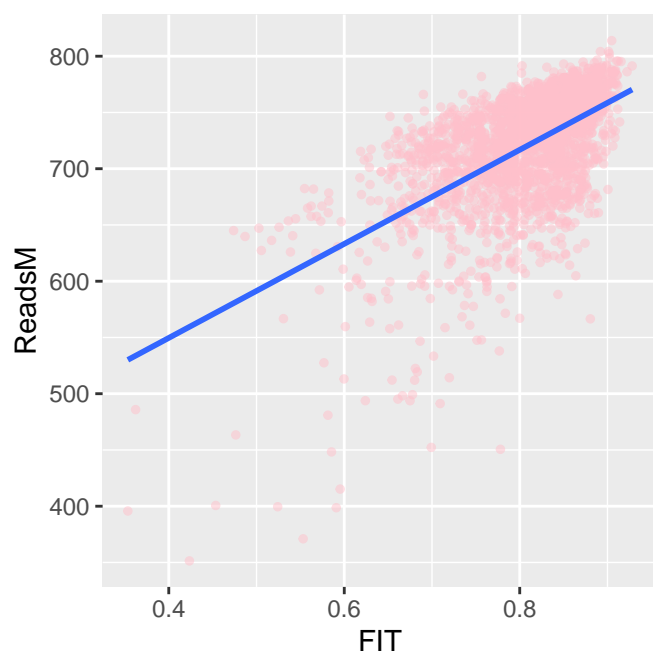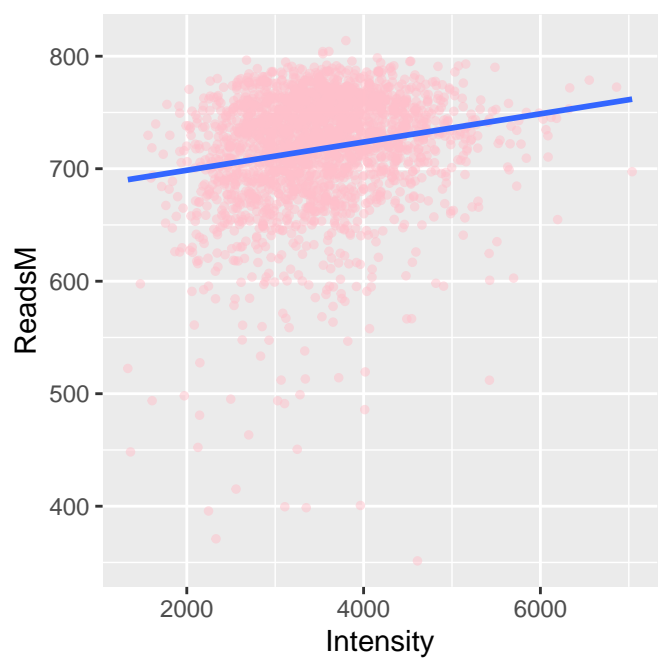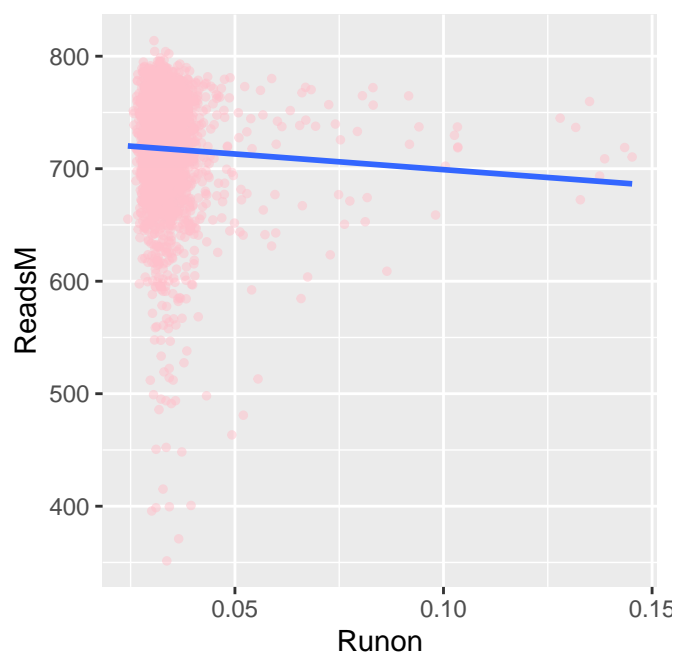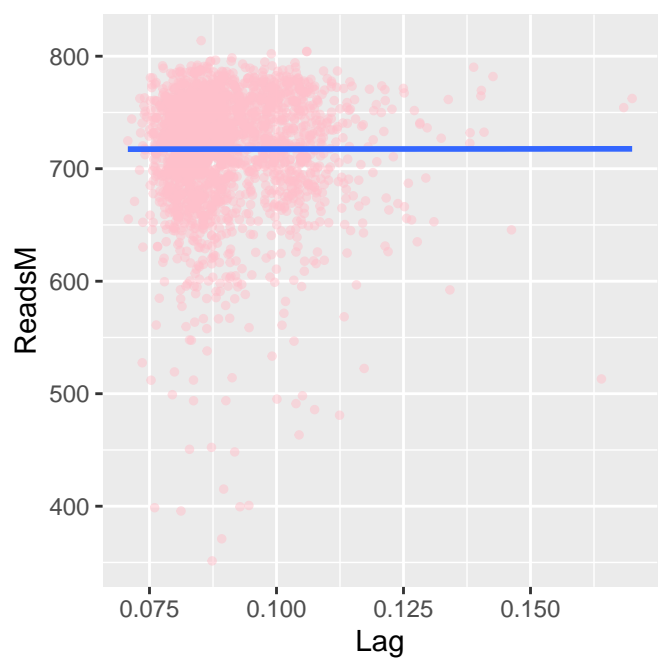

Supplement: Supplementary file 1 — Additional file 1: Figure S1. The correlation of each metric with yield (ReadsM). The linear regression line is shown in blue. [file 13040_2019_209_MOESM1_ESM.pdf]

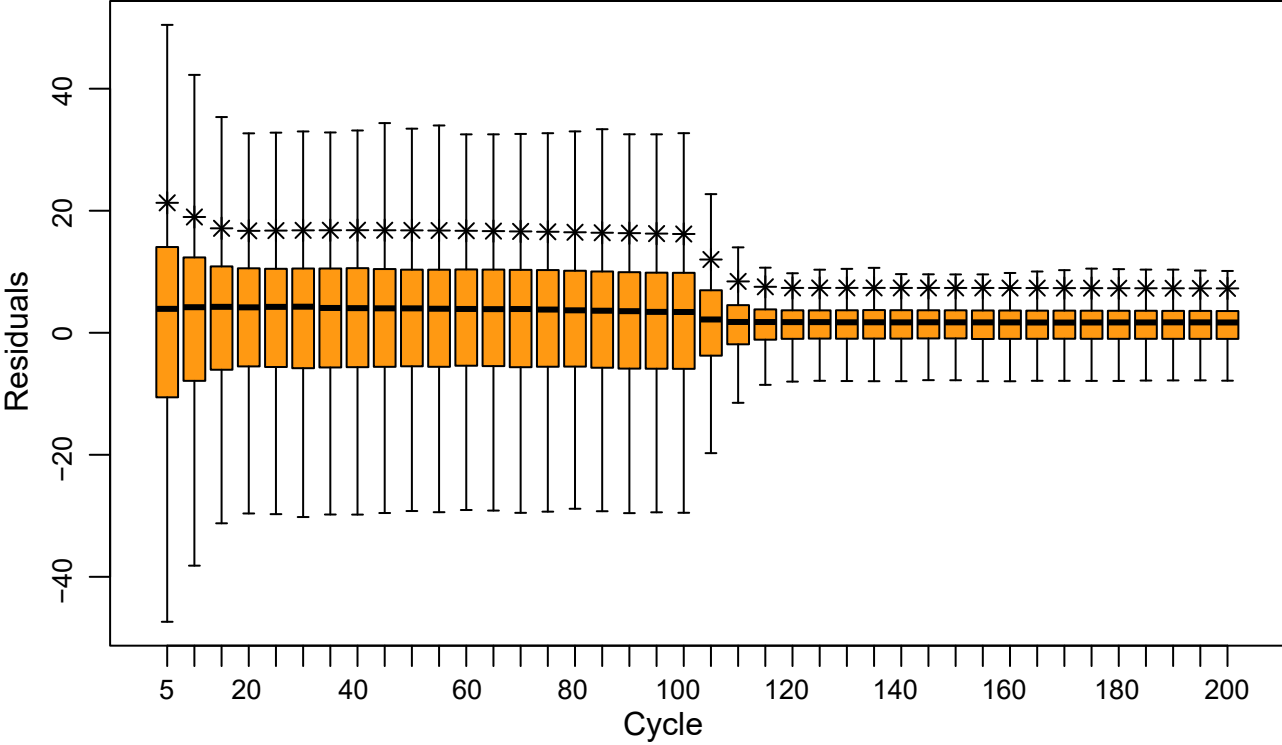

Supplement: Supplementary file 2 — Additional file 2: Figure S2. Residual deviation of the LR model every 5 cycles. The box plot displays the residuals of all 40 LR models; each box shows the median and first and third quartiles, and a star indicates the standard deviation. [file 13040_2019_209_MOESM2_ESM.pdf]
